# SPECT/CT imaging in thyroid eye disease: an emerging role in assessing sub-clinical activity

**DOI:** 10.3389/fopht.2026.1792332

**Published:** 2026-08-20

**Authors:** Kelsey A. Roelofs, Kimberly M. Papp, Kate S. Lim, Ezekiel Weis

**Affiliations:** 1 Department of Ophthalmology and Visual Sciences, University of Alberta, Edmonton, AB, Canada; 2 Department of Ophthalmology, University of British Columbia, Vancouver, BC, Canada; 3 Department of Surgery, Faculty of Medicine, University of Calgary, Calgary, AB, Canada

**Keywords:** clinical activity score (CAS), graves orbitopathy, orbital imaging, SPECT/CT, thyroid eye disease, thyroid orbitopathy

## Abstract

Thyroid eye disease (TED) diagnosis is made based on the presence of a constellation of clinical, laboratory, and radiologic findings. Currently, computed tomography (CT) and magnetic resonance imaging (MRI) are the most commonly used imaging modalities; however, they provide indirect indicators of underlying inflammation, resulting in limited specificity. Our narrative review explored the role of Single Photon Emission Computed Tomography (SPECT)/CT in TED with the goal of enhancing our ability to correlate structure with function in TED. A non-systematic narrative search of major electronic databases was conducted to identify literature on SPECT/CT in TED, using targeted selection and thematic narrative synthesis to analyze the findings. Technetium-99m diethylenetriaminepentaacetic acid (^99m^Tc-DTPA) is the most common tracer used in SPECT/CT and it undergoes extravasation in the presence of inflammatory mediators. The uptake ratio (UR) of this tracer in orbital tissues can be calculated and compared with a reference tissue to provide a way of direct spatial visualization of function and corresponding structure. The UR was consistently higher in patients with a clinical activity score (CAS) ≥ 3. The UR was also consistently elevated in those identified as having active TED on MRI, indicating good correspondence. Lastly, the UR was reduced in the extraocular muscle after introducing triamcinolone injection, highlighting the usefulness of SPECT/CT in identifying anatomic disease activity and guiding treatment among a cohort of CAS-negative patients. While the quantitative calculation of the ^99m^Tc-DTPA UR provides an objective metric for evaluating active disease, its clinical use in TED is still limited by substantial inter-study variability and intrinsic technical limitations. Monitoring UR may be better suited for monitoring each patient’s progression within the same institution.

## 1 Introduction

The diagnosis of thyroid eye disease (TED) is based upon the presence of a constellation of clinical, laboratory, and radiologic findings. Orbital imaging can provide the clinician with valuable insights throughout the diagnosis and management of TED, from predicting response to various treatments to creation of a customized surgical plan. A variety of imaging modalities and techniques can be utilized in TED, most commonly including computed tomography (CT) with or without contrast and/or magnetic resonance imaging (MRI) (1); however, as our biological understanding of TED continues to advance, we are prompted to consider how imaging can be leveraged to further individualize patient care.

Several characteristics on MRI have been correlated with the active phase of TED, including increased T2- based signal intensity ratio (SIR), prolonged T2 relaxation time (2, 3), higher wash-in rate of extraocular muscle (EOM) contrast enhancement (4), lower fractional anisotropy (FA) (5), and higher apparent diffusion coefficient (ADC) of the lacrimal gland (6, 7) or EOMs (8). While these features can provide useful insights, they are all *indirect* indicators of underlying inflammation, and this can result in limited specificity.

With the goal of enhancing our ability to correlate structure with function in TED, more nuanced neuroimaging techniques including structural, functional, diffusion and metabolic MRI (9) and single-photon emission CT (SPECT) have been investigated. SPECT/CT is a functional nuclear medicine imaging technology that allows for spatial overlay of anatomic (CT) and functional (SPECT) images. The purpose of this narrative review is to summarize the use of SPECT/CT in TED, highlight its ability to quantify disease activity, discuss correlation with other orbital imaging modalities, and touch on its potential role for individualizing treatment algorithms in TED.

## 2 Methodology

A narrative literature search was conducted to identify literature evaluating the diagnostic, prognostic, and management utility of SPECT/CT in TED. Major electronic databases, including PubMed, MEDLINE, and Embase, as well as reference lists of relevant articles and comprehensive imaging reviews, were searched without restriction on publication date up to November 2025. Search queries incorporated combinations of Medical Subject Headings (MeSH) terms and relevant keywords, including terms such as “single-photon emission-computed tomography”, “SPECT-CT”, “thyroid eye disease”, “Graves’ ophthalmopathy”, “thyroid-associated ophthalmopathy”, and “orbital imaging”. Literature selection was targeted toward studies directly addressing primary clinical questions regarding the utility of SPECT/CT in TED. Studies were included if they evaluated:

1. SPECT/CT clinical applications in TED (including technetium-99m-labelled diethylenetriaminepentaacetic acid (^99m^Tc-DTPA));
2. Correlations between imaging findings and clinical parameters, including clinical activity score (CAS), disease staging/activity, and dysthyroid optic neuropathy (DON);
3. Comparisons between SPECT/CT and other imaging modalities (e.g., CT, MRI); or
4. Predictive value regarding response to medical therapies (e.g., glucocorticoids) or surgical interventions (e.g., orbital decompression).

Studies were excluded if they lacked full-text manuscripts, evaluated standalone SPECT or standard CT without hybrid fusion imaging, or focused exclusively on non-thyroid eye disease pathologies.

Given the anticipated heterogeneity in patient cohorts, radiotracers, and quantitative imaging metrics (e.g., target-to-background ratios or orbital uptake values), a formal meta-analysis was not performed. Data were instead synthesized narratively and organized by primary clinical themes: clinical activity/staging correlations, comparative imaging characteristics, and treatment response prediction.

The terms thyroid eye disease and Graves’ orbitopathy/ophthalmopathy were used interchangeably across many studies included in our review. We use thyroid eye disease (TED) as the primary term to encompass all of these different nomenclatures.

## 3 SPECT/CT

### 3.1 SPECT/CT technical principles and mechanisms for identifying inflammation

SPECT/CT utilizes gamma-emitting radioisotopes, most commonly, ^99m^Tc-DTPA (10). Compared to the radiotracers utilized in positron emission tomography (PET), ^99m^Tc-DTPA employed in SPECT has a longer half-life, allowing for broader clinical uses and improved cost-effectiveness. In addition, there is lower radiation exposure for the patient (11). While other tracers exist and have been applied to TED (e.g. ^99m^Tc-HYNIC-TOC and ^99m^Tc-P829) (12–14), only ^99m^Tc-DTPA was utilized in studies captured by our literature review; therefore, we will focus our remaining discussion on ^99m^Tc-DTPA.

^99m^Tc-DTPA is an extracellular, non-protein bound radiotracer. In the presence of inflammatory mediators, capillary permeability increases, resulting in extravasation of ^99m^Tc-DTPA (15, 16). Heightened permeability also increases the extracellular volume available for ^99m^Tc-DTPA to collect, and slowed clearance of the radiotracer from the inflamed interstitium further compounds its accumulation. The emitted gamma rays are then detected by a camera system and reconstructed to produce an image that can be fused with the corresponding anatomic CT, allowing *direct* spatial visualization of function and corresponding structure (10). SPECT/CT images can be analyzed and an uptake ratio (UR) can be calculated that compares the measurement of the radiotracer in orbital soft tissues to a reference tissue, most commonly the occipital lobe (17). Unfortunately, while being a key metric, there is a lack of validated methods and reference ranges for URs in the setting of TED. Two authorship groups appear to have reached a consensus (18, 19) and have detailed these methods, with Li et al. advocating for measuring the UR of the most severely affected EOM as the strongest reliable predictor of disease activity (18).

While the quantitative calculation of the ^99m^Tc-DTPA UR provides an objective metric for evaluating active disease, its clinical interpretation is heavily dictated by practical imaging determinants. Understanding these factors is critical, as substantial inter-study variability limits direct numerical cross-comparisons between published diagnostic thresholds:

First, the temporal window between radiopharmaceutical injection and SPECT/CT acquisition directly dictates the tracer’s distribution profile (20). Because ^99m^Tc-DTPA relies on passive diffusion through compromised capillary walls into the expanded extracellular space of the active orbit, early scans reflect vascular hyperperfusion, whereas delayed acquisitions capture true tissue edema. Variations in protocol timing across centers inherently skew absolute UR values.

Furthermore, the Region-of-Interest (ROI) Delineation is a strategy used to isolate target (21)tissues, ranging from manual 2D ROI profiling of the highest-intensity focal point to semi-automated 3D Volume-of-Interest (VOI) segmentation of entire EOM bellies, introducing operator dependency (21). Additionally, protocols utilizing maximum pixel values, UR_max_, yield significantly elevated thresholds compared to those relying on mean spatial values UR_mean_ (17, 19).

Importantly, the occipital lobe is a widely accepted internal reference standard because its intact blood-brain barrier (BBB) excludes ^99m^Tc-DTPA, yielding a stable, low-background signal that isolates the pathologically hyper-permeable orbital tissue (22). However, slight variations in background ROI sizing or axial slice alignment relative to the optic nerve level create baseline fluctuations that directly influence the calculated ratio.

Consequently, while absolute UR cutoffs vary across the literature, the intra-patient and longitudinal utility of the metric remains robust when imaging parameters are standardized. Therefore, UR can serve as an additional modality of investigation when uncertainties arise with TED management based only on clinical signs and symptoms, providing an objective functional counterpoint to subjective clinical assessments such as the CAS.

### 3.2 Correlation with SPECT/CT and clinical activity score

Accurate identification, and ideally quantification, of the degree of inflammation in TED is important from both a clinical and research perspective. A CAS ≥ 3 is commonly employed as the definition of active disease; however, CAS contains several subjective metrics, and there is a subset of patients with TED who have evidence of disease activity and/or progression yet are “CAS negative”. Moreover, CAS can yield false positive conclusions when signs are secondary to residual congestion despite resolution of active inflammation. Nonetheless, CAS is the most broadly used scale describing TED activity and thus, despite its flaws, understanding the correlation between SPECT/CT and CAS is important in contextualizing the utility of this imaging technology.

In a retrospective study of 67 patients with TED, Li et al. noted that patients with CAS ≥ 3 have significantly higher UR on SPECT/CT with ^99m^Tc-DTPA within the EOMs and lacrimal gland compared to their inactive (CAS < 3) counterparts (18). Jiang et al. prospectively evaluated SPECT/CT on a large cohort of patients (206 patients with TED and 14 healthy controls), similarly concluding that active TED corresponded to higher UR on SPECT/CT (19). Liu et al. went on to create three predictive models: SPECT/CT, clinical, and clinical-radiomics (23). They concluded that incorporation of texture features from SPECT and CT data improved the model’s performance for identifying active TED, with areas under the receiver operating characteristic (AUROC) curve of 0.94 and 0.91 in the training and test sets, respectively (23). Detailed information on the parameters compared in each study can be found in **Table 1**.

**Table 1:** Clinical diagnostic performance and quantitative parameters of ^99m^Tc-DTPA imaging in assessing thyroid eye disease activity.

Author, year, study design	Sample size (n)	Comparator or ground truth	Key quantitative findings (active vs. inactive)	Diagnostic performance metrics (95% CI)	Limitations
Li and Liu. (2023) Retrospective (18)	61 patients (23 active, 38 inactive)	CAS (active if ≥ 3/7)	**Significant UR elevation in active states across all EOMs (p < 0.01)** • SR: 4.91 +/- 1.63 vs. 4.02 +/- 1.15 • IR: 5.27 +/- 1.49 vs. 4.25 +/- 1.06 • MR: 5.56 +/- 1.70 vs. 4.36 +/- 1.17 • LR: 4.98 +/- 1.31 vs. 4.21 +/- 1.07 • UR_max_: 6.18 +/- 1.95 vs. 4.85 +/- 1.11	**AUROC:** • UR_max_: 0.803 (0.690, 0.915) • Lacrimal gland: 0.786 (0.706, 0.867) • EOM model: 0.730 (0.686, 0.0775) • Optic nerve: 0.635 (0.537, 0.733) (All p = 0.000)	• Cohort lacks a homogenous severity distribution (skewed toward CAS 3-4; few severe cases). • Lacks longitudinal evaluation to track progression. • Small sample size
Jiang et al. (2021) Retrospective (19)	206 patients (440 eyes: 195 active, 217 inactive) + 14 controls	CAS (active if ≥ 3/7)	**Significant elevations in active eyes (p < 0.003 except LR):** • U_max_: 12.31 +/- 2.27 vs. 9.76 +/- 2.03 • T_max_: 6.9 +/- 2.0 mm vs. 5.2 +/- 1.7 mm	**AUROC Models:** • Clinical alone: 0.67 • Clinical + CAS ≥ 3: 0.78 • Clinical + U_max_: 0.83 • Combined model: 0.85	• No uniform consensus on the objective definition of treatment response. • Partial volume effect compromises quantitative SPECT accuracy in small EOM structures.
Galuska et al. (2002) (20) Prospective	14 patients (28 eyes) Includes 9 control	1.0T MRI T2 times (Active ≥ 70 ms)	Orbital-to-brain (OR/B) ratio significantly higher in active orbits (8.30 +/- 2.08 vs. 6.4 +/- 1.17, p < 0.05). ^99m^Tc-DTPA uptake directly correlated with MRI activity scores.	Not reported	• Small sample size • Inferior structural resolution of standard SPECT compared to MRI
Szumowski et al. (2019) (26) Prospective	23 patients Includes 9 controls	Consensus of CAS (≥ 3) and 3T MRI (T2/T1+ signal)	Evaluated head-to-head diagnostic utility of SPECT/CT vs. MRI vs. CAS (p < 0.05 for imaging vs. CAS).	**SPECT/CT (95% CI):** • Sensitivity: 0.93 (0.83, 0.99) • Specificity: 0.89 (0.58, 0.99) • Accuracy: 0.91 (0.92, 0.98) **3T MRI:** • Sensitivity: 0.93 (0.81, 0.98) • Specificity: 0.78 (0.55, 0.89) • Accuracy: 0.87 (0.58, 0.94) **CAS:** • Sensitivity: 0.86 (0.51, 0.92) • Specificity: 0.56 (0.29, 0.56) • Accuracy: 0.74 (0.63, 0.88)	• Small sample size • Defining the “ground truth” via a consensus combination of the tested indices inherently biases individual evaluations.

CAS, clinical activity score; UR, uptake ratio; SR, superior rectus; IR, inferior rectus; MR, medial rectus; LR, lateral rectus; EOM, extraocular muscles; UR_max,_ max ratio among the four EOMs); LG, lacrimal gland; AUROC, area under the receiver operating characteristic curve; CI, confidence interval; T_max,_ maximum thickness among the four EOMs; U_max,_ maximum uptake ratio among the four EOMs; Tc-DTPA, 99m technetium-labelled diethylene triamine pentaacetic acid; SPECT, single-photon emission computed tomography; MRI, magnetic resonance imaging.

Currently, several studies have explored the use of artificial intelligence (AI) in different imaging modalities for TED (9). There are two primary literature that specifically investigated SPECT/CT’s role in diagnostic efficiency of TED (23, 24). Yao et al. developed a deep learning (DL)-based AI model in their retrospective study of 478 patients with TED, and their model accurately distinguished active versus inactive disease (sensitivity 84.63%, specificity 83.87%) (24). Similarly, Liu et al. explored whole-orbit radiomics signatures, extracting high-throughput texture features to assess diagnostic efficiency in different imaging modalities (23). They used a clinical model and a combined model based on Rad-score and clinical parameters combined (23). Although these studies utilized methodologies with different strengths, they both demonstrated high sensitivity: the DL automated segmentation framework excels at tracking rapid macro-volumetric changes in fat and EOM, while the radiomics approach successfully isolates subtle micro-structural tissue variations (such as edema versus fibrosis). Reassuringly, active TED was universally associated with detectable alterations in image texture and tissue metrics compared to inactive controls. The ability of machine learning to isolate these active pathological features stands as a robust qualitative finding. However, it is still premature to translate this qualitative trend into rigid quantitative claims such as definitive diagnostic thresholds, or absolute prediction accuracies. **Table 2** features the AI method and performance metrics used in each AI-related study.

**Table 2:** Application of machine learning, deep learning, and radiomics models for activity and severity assessment in thyroid eye disease.

Author, year, study design	Sample size (n)	Imaging modality & data source	AI method, architecture, & clinical task	Key performance metrics	Methodological limitations
Liu et al. (2024) (23) Retrospective	173 patients	^99m^Tc-DTPA orbital SPECT/CT vs. CT only Multi-center study	Whole-orbit radiomics signature extracting high-throughput texture features to assess diagnostic efficiency in different imaging modalities. Used clinical model and a combined model based on Rad-score + clinical parameters.	AUROC: 0.91	• Radiomics features are highly sensitive to subtle variations in manual region-of-interest (ROI) boundary definitions. • Potential overfitting to retrospective training datasets.
Yao et al. (2023) (24) Retrospective cohort	478 patients (956 eyes: 475 active, 481 inactive)	^99m^Tc-DTPA orbital SPECT/CT vs. CT only vs. CT + EOM masks vs. SPECT/CT + EOM masks	Deep learning framework combining SV-Net (for automated EOM segmentation) with a CNN (for binary activity assessment) to assess diagnostic efficiency.	AUROC: 0.89	• Retrospective data design introduces potential selection bias • Deep learning models lack direct transparency (“black box”) regarding specific pixel-level feature weighting.
Zhang et al. (2026) (25) Systematic review	2 studies specific to nuclear imaging in TED (including Yao and Liu)	Heterogenous nuclear imaging and multi-modality datasets	Systematic synthesis of AI applications in TED imaging, focusing on “enhanced detection” of soft-tissue changes.	N/A	• High study heterogeneity across the available literature limits meta-analysis capacity • Small number of published validation cohorts globally

IVGC, intravenous glucocorticoid; AUROC, area under the receiver operating characteristic curve; CT, computed tomography; SV-Net, Support Vector Network; CNN, Convolutional Neural Network; EOM, End-of-Month; AI, artificial intelligence; TED, thyroid eye disease.

### 3.3 Correlation of SPECT/CT with other orbital imaging modalities utilized in TED

Unlike other imaging modalities that use indirect markers of inflammation, SPECT/CT facilitates a more direct measurement of the biologic processes at hand, presuming they relate to the inflammatory cascade. In 14 patients (28 orbits) with evidence of active TED on MRI, defined as the number of recti muscles with a T2 relaxation times of > 70 ms, UR on SPECT/CT was similarly increased (22). Szumowski et al. utilized a combination outcome of CAS, MRI (T2 signal increase and T1 signal strengthened), and SPECT/CT (increased accumulation of radiotracer) to determine disease activity (26). In their study, a positive result of two out of three tests (CAS, MRI, or SPECT/CT) served as their definition of active TED. Regarding validity measures, they found similar sensitivity of SPECT/CT and MRI for identifying active disease (0.93) but concluded that SPECT/CT had superior positive predictive value (0.93), negative predictive value (0.89), and had higher specificity(0.89) than either MRI (0.78) or CAS (0.56) for identifying active disease (26). It is important to recognize that while these values favor the use of SPECT/CT, they were based on a single, relatively small study that defined the ‘ground truth’ using a consensus combination of the tested indices.

### 3.4 Ability of SPECT/CT to predict response to treatment

Liu et al. specifically looked at a group of 102 CAS-negative patients (CAS <3) with findings of disease activity on SPECT/CT as evidenced by high extraocular muscle(s) uptake of ^99m^Tc-DTPA (27). They evaluated findings on SPECT/CT pre- and post- peribulbar triamcinolone injection. Moreover, selection of the peribulbar injection site was based on the spatial findings on SPECT/CT. For instance, in a patient with ^99m^Tc-DTPA only in the superior rectus, the injection was placed in the superomedial quadrant of the orbit. They observed a decrease in UR on SPECT/CT following triamcinolone injection and an improvement in symptoms following periocular triamcinolone injection (27). The authors concluded that SPECT/CT was useful in identifying anatomic disease activity and guiding treatment amongst a cohort of CAS-negative patients (27).

Jiang et al. similarly identified that SPECT/CT was useful in predicting response to local glucocorticoids, concluding that patients with a higher maximum uptake of ^99m^Tc-DTPA were more likely to respond to treatment (19). These authors found UR to be an independent predictor of response even when CAS and other clinical characteristics were accounted for (19). Efficacy indicators and imaging changes following intravenous triamcinolone injection in TED can be found in **Table 3**.

**Table 3:** Efficacy indicators and imaging changes following targeted therapeutic interventions in active thyroid eye disease.

Author, year, study design	Sample size (n)	Therapeutic intervention	Definition of response/ground truth	Post-treatment objective changes (imaging & clinical)	Study limitations
Liu et al. (2020) (23) Retrospective cohort	64 patients (89 eyes) Includes 19 controls	Local peribulbar triamcinolone vs. untreated control group.	Baseline clinical activity score CAS < 3 combined with high tracer uptake on SPECT/CT (identifying sub-clinical posterior inflammation).	**Clinical:** CAS decreased significantly at 6 months (0.4 +/- 0.5 vs. 1.0 +/- 0.9; p < 0.001) **Imaging:** At 3 months, significant reductions occurred in IR and MR muscle thickness (p < 0.001) alongside greater mean reductions in tracer uptake (IR: p = 0.001; MR: p = 0.011) compared to controls.	• Lack of demonstrable functional improvement (e.g., exophthalmos or motility) despite clear reduction in tissue edema and muscle size • Retrospective nature
Jiang et al. (2021) (19) Treatment efficacy sub-cohort	86 eyes total (56 responder, 30 non-responder)	Pneumatic Galvanic Therapy (PGT)	Clinical/imaging response classifications based on post-therapy follow-up evaluations.	**Responders (56 eyes):** EOM thickness, UR of each EOM, T_max_, and U_max_ dropped significantly following therapy (all p < 0.01) **Non-responders (30 eyes):** Quantitative UR parameters remained statistically unchanged (all p > 0.05)	• There is no uniform consensus across the global ophthalmic community regarding the objective definition of “treatment response” in TED. • The partial volume effect inherently compromises the absolute quantitative accuracy of SPECT imaging in small structures like EOMs.

CAS, clinical activity score; SPECT, single-photon emission computed tomography; CT, computed tomography; IR, inferior rectus; MR, medial rectus; EOM, extraocular muscles; T_max,_ maximum thickness among the four EOMs; U_max,_ maximum uptake ratio among the four EOMs; UR, uptake ratio; TED, thyroid eye disease.

## 4 Discussion

This review has amalgamated existing literature on the use of SPECT/CT in TED, highlighting advantages and caveats of using such imaging modality in TED. ^99m^Tc-DTPA SPECT/CT quantifies retrobulbar inflammation through uptake ratio and corrected maximum standardized uptake value (SUV_max_), which correlate significantly with CAS and MRI T2 relaxation time scores (21, 22). A corrected SUV_max_ threshold of 10.440 yielded 70.7% sensitivity and 82.1% specificity for distinguishing active from inactive disease (21).

### 4.1 Methodological determinants of uptake ratio and inter-study variability

When synthesizing the diagnostic utility of ^99m^Tc-DTPA across the included literature, the lack of a universal, standardized threshold for the UR emerges as a prominent theme. The absolute cutoffs and calculated ratios vary notably between key studies. For instance, foundational work by Galuska et al. established early validation by correlating orbital-to-brain count ratios (OR/B) directly with MRI T2 relaxation times (22). However, subsequent studies captured in our narrative review, such as Szumowski et al. and Liu et al., utilized distinct acquisition workflows, and varying radiotracer timing, and individual inclusion boundaries, yielding inherently different numerical baselines (26, 27).

Rather than exposing a flaw in the modality, this variability highlights the significant impact of technical and procedural methodology on quantitative nuclear imaging. The time elapsed between tracer injection and image acquisition varies across these historical and modern cohorts. Because ^99m^Tc-DTPA accumulation relies on the passive kinetics of interstitial leakage into inflamed orbital tissue, a center scanning at an earlier time point captures vascular perfusion, whereas a delayed scan measures true tissue edema (28, 29). Additionally, the mathematical definition of the UR itself is highly sensitive to operator methodology. Studies drawing a maximum intensity pixel ROI UR_max_ naturally report higher absolute values than those employing a mean volumetric approach UR_mean_ across the extraocular muscles (17, 19, 21). Furthermore, minor variations in defining the background reference slice within the occipital lobe introduce baseline shifts that alter the final ratio. These variations explain why Szumowski et al. achieved a remarkable specificity of 0.89, while Liu et al. successfully utilized the modality to identify active posterior orbital disease in patients who presented with a misleadingly low CAS (< 3) (26, 27). The clinical takeaway is clear: the absolute numerical values of the UR cannot simply be extrapolated from one study to another.

Consequently, while the variability in the literature mandates caution when attempting meta-analyses or establishing universal diagnostic cutoffs. When parameters are standardized within a single institution, the UR remains a robust, objective metric. It avoids some of the limitations of subjective clinical scoring systems, providing a highly reproducible means to map bilateral asymmetry and track real-time longitudinal therapeutic responses in patients with TED.

### 4.2 Qualitative consistency vs. quantitative uncertainty in SPECT-CT

Across all included studies, a consistent qualitative signal emerged. Active TED was universally associated with elevated UR compared to inactive controls or healthy tissues. This consistent trend validates the underlying physiological mechanism that active inflammation and subsequent tissue edema reliably drive tracer accumulation. The ability of an elevated UR to reflect active disease processes remains a highly reproducible qualitative finding across diverse patient cohorts.

However, translating this reliable qualitative signal into definitive quantitative metrics, such as universal diagnostic thresholds, remains challenging. The magnitude and clinical certainty of specific numerical values are not yet established due to small and heterogenous cohorts and methodological variances as previously detailed. Consequently, specific quantitative claims such as exact optimal UR thresholds or precise specificity margins over other modalities like MRI must be interpreted with caution, as they rest on methodologically varied frameworks.

### 4.3 Limitations of SPECT/CT as a modality

SPECT/CT imaging has intrinsic technical limitations. SPECT alone provides poor spatial resolution for surgical planning (1, 30). The CT component helps but does not match the soft tissue resolution of MRI for distinguishing edema from fibrosis (1, 30). Furthermore, the lack of standardized cut-off values for SUV or UR to define disease activity and methodology for activity assessment limits its applicability in the real clinical setting. Additionally, the concerns around the reproducibility of UR, showing reduced interobserver consistency (ICC = 0.632), limits its standalone use (21, 30). Lastly, there’s still limited validation of its use as most studies are single-center with relatively small sample sizes. Multicenter validation studies are needed before widespread clinical adoption.

Furthermore, there are other practical limitations to SPECT/CT imaging. Firstly, ^99m^Tc is currently the most common radiotracer used in diagnostic imaging, and concerns have been raised since the early 2010s about the potential for supply shortage (31, 32). As another pragmatic limitation, CT imaging results in radiation exposure to the patient, in contrast to MRI. Fortunately, SPECT/CT has remained an overall cost-efficient imaging modality (11), and the radiation dose from SPECT/CT imaging is acceptably low (26).

Perhaps one of the most valuable insights gained from SPECT/CT imaging in the setting of TED is the correlation of structure with function, lending insights surrounding the biological process at hand; however, the literature drawing these conclusions is limited by the fact that there is no consensus regarding the gold standard for defining TED activity. Multiple studies have used CAS as the ground truth, yet it is well recognized that there are subsets of patients with active disease who are CAS negative. Moreover, orbital congestion can lead to a falsely positive CAS, making this score a relatively poor predictor of response to treatment. While it is possible that SPECT/CT may have greater specificity for identifying patients with sub-clinical inflammation than either CAS or MRI, drawing this conclusion is challenging when the ground truth remains difficult to define. Future directions to address this issue may expand on and replicate strategies akin to Szumowski et al., whose study had a modest number of participants (n = 23) but employed an innovative multimodal system of defining active TED as two of three conditions positive between CAS, SPECT/CT, and MRI, recognizing the risk of false negatives when using only a single measurement (26). Furthermore, establishing a UR threshold that reliably defines activity would provide a crucial benchmark for future investigations.

### 4.4 Limitations of this review

While this review provides a comprehensive overview of the clinical values and disadvantages of using SPECT/CT in TED, there are several limitations. The existing literature surrounding SPECT/CT in TED is predominantly comprised of retrospective, single-center studies that are modest in sample size. Furthermore, several studies use CAS as the ground truth, a fundamental limitation as has been discussed previously. Additionally, our review utilized a narrative search and selection strategy. While this approach lacks the rigid query strings and quantitative pooling of a formal systematic review or meta-analysis, it was deliberately chosen to accommodate the broad, heterogenous nature of the available literature and capture clinically pragmatic applications of SPECT/CT in TED. Nevertheless, this non-systematic approach and targeted study selection may introduce inherent database or publication biases. However, given the significant heterogeneity in patient cohorts, varying quantitative imaging metrics, and the diverse radiotracers utilized across the current SPECT/CT literature, this narrative approach was the most appropriate method to qualitatively contextualize and map the evolving clinical landscape of thyroid eye disease imaging. With these in mind, the conclusions drawn from this review should be taken as a comprehensive summary of the existing body of literature at this point in time.

### 4.5 Summary

In summary, SPECT/CT has the capability to provide anatomically localized, quantitative analysis of the inflammatory activity in patients with TED. Future research evaluating the ability of SPECT/CT to predict response to various treatments in TED may be valuable in enhancing the personalization of our approach to management of this heterogeneous autoimmune condition. Similarly, utilizing more advanced, or combined methods of quantifying activity (such as the methodology employed by Szumowski 2019) (26) may reduce the challenges regarding the ground truth for activity, allowing for more accurate assessment.
